# Malaria during pregnancy and newborn outcome in an unstable transmission area in Brazil: A population-based record linkage study

**DOI:** 10.1371/journal.pone.0199415

**Published:** 2018-06-21

**Authors:** Jamille Gregório Dombrowski, Rodrigo Medeiros de Souza, Natércia Regina Mendes Silva, André Barateiro, Sabrina Epiphanio, Lígia Antunes Gonçalves, Claudio Romero Farias Marinho

**Affiliations:** 1 Department of Parasitology, Institute of Biomedical Sciences, University of São Paulo, São Paulo, Brazil; 2 Multidisciplinary Center, Federal University of Acre, Acre, Brazil; 3 Department of Clinical and Toxicological Analyses, School of Pharmaceutical Sciences, University of São Paulo, São Paulo, Brazil; Université Pierre et Marie Curie, FRANCE

## Abstract

**Background:**

Malaria in pregnancy (MiP) is one of the major causes of mortality and morbidity in tropical regions, causing maternal anemia, intrauterine growth retardation, preterm birth, and low birth weight (LBW). The integration of the information systems on pregnancy and malaria could prove to be a useful method of improved decision making for better maternal-child health.

**Methods:**

A population-based observational study acquired information retrospectively from all live births that occurred between 2006 and 2014 in Cruzeiro do Sul (Acre, Brazil). Social and clinical data of the mother and newborn was extracted from the Information System of Live Births. Malaria episodes information was obtained from the Brazilian Epidemiological Surveillance Information System Malaria. A deterministic record linkage was performed to assess malaria impact on pregnancy.

**Results:**

The studied population presented a malaria incidence of 8.9% (1283 pregnant women infected), of which 63.9% infected by *Plasmodium (P*.*) vivax*. Reduction of newborn birth weight at term (small for gestational age (SGA) and LBW) has been found associated with *P*. *vivax* infection during pregnancy (SGA—OR 1.24, 95% CI 1.02–1.52, p = 0.035; term LBW—OR 1.39, 95% CI 1.03–1.88, p = 0.033). Additionally, *P*. *falciparum* infection during pregnancy has been found to be associated with preterm births (OR 1.54, 95% CI 1.09–2.18, p = 0.016), which is related with late preterm births (OR 1.59, 95% CI 1.11–2.27, p = 0.011).

**Conclusions:**

Despite the decrease of malaria cases during the evaluation period and regardless of *Plasmodium* species, we present evidence of the deleterious effects of MiP in a low transmission area in the Amazonian region.

## Introduction

Malaria is a severe and potentially fatal parasitic disease that constitutes a major public health issue, being one of the greatest causes of mortality in tropical regions [1]. Pregnant women are particularly vulnerable to malaria infection and it is estimated that 125 million women are at risk of malaria in pregnancy (MiP) each year [1,2]. Malaria can be devastating for both mother and fetus, leading up to 10,000 maternal and 75,000 to 200,000 child deaths each year [3]. Maternal malaria presents a significant impact on the neonates, being associated with increased risk of spontaneous abortion, stillbirth, premature delivery, fetal death, low birth weight (LBW) and fetal/child development retardation in malaria-endemic countries [1,3].

LBW reflects an intra-uterine growth retardation (IUGR) and preterm delivery, which are compelling indicators of infant morbidity [3–6]. LBW has been linked to infant mortality and poor cognitive development, and the occurrence of non-communicable diseases later in life [6,7]. In fact, LBW due to malaria is related with up to 100,000 infant deaths each year in endemic countries [8,9]. These adverse birth outcomes have been extensively associated with *P*. *falciparum* infection during pregnancy. In contrast to *P*. *falciparum*, the *P*. *vivax* burden in pregnancy is less well described, and have been described as having less impact in the newborn [3,10]. Though, recent studies have presented the two species as similar threats to the mother and fetus [11,12]. Despite the efforts to reduce malaria the prevalence of these adverse birth outcomes remains high.

Therefore, it is crucial to have an efficient epidemiological surveillance of malaria during pregnancy. The linkage of two or more health public surveillance record databases with shared variables presents an important and effective strategy to plan preventive measures. Currently, most of the malaria-endemic countries have malaria public surveillance record databases since it is compulsory notification disease. This will contribute to the identification of epidemics and areas most affected. Thus, allowing to direct and intensify the control and preventive measures to the affected communities, and reduce negative birth outcomes [13]. In fact, due to the potential assemble with other information systems it can be recognized as an important tool for research [14–16].

In 2003, the Brazilian Epidemiological Surveillance Information System (SIVEP)-Malaria was implemented to systematize the flow and quality of the information on malaria. This system gathers information on malaria morbidity according to gender, age, *Plasmodium* species, site of residence, the probable site of infection, treatment, and pregnancy status [17]. Another essential Brazilian information system of national coverage is the Information System of Live Births (SINASC), implemented in 1990. This system collects and systematizes information on maternal, pregnancy, delivery and newborn data [18],[19]. Linkage of record databases is still scarcely used in Brazil, despite being an easy to perform technique with low operational cost. Here we present the first study that evaluates the association between MiP and adverse birth outcomes in the Brazilian Amazonian region for nine years (2006–2014), using information obtained through the linkage of SINASC and SIVEP-Malaria.

## Materials and methods

### Study design and data collection

**Fig 1:**
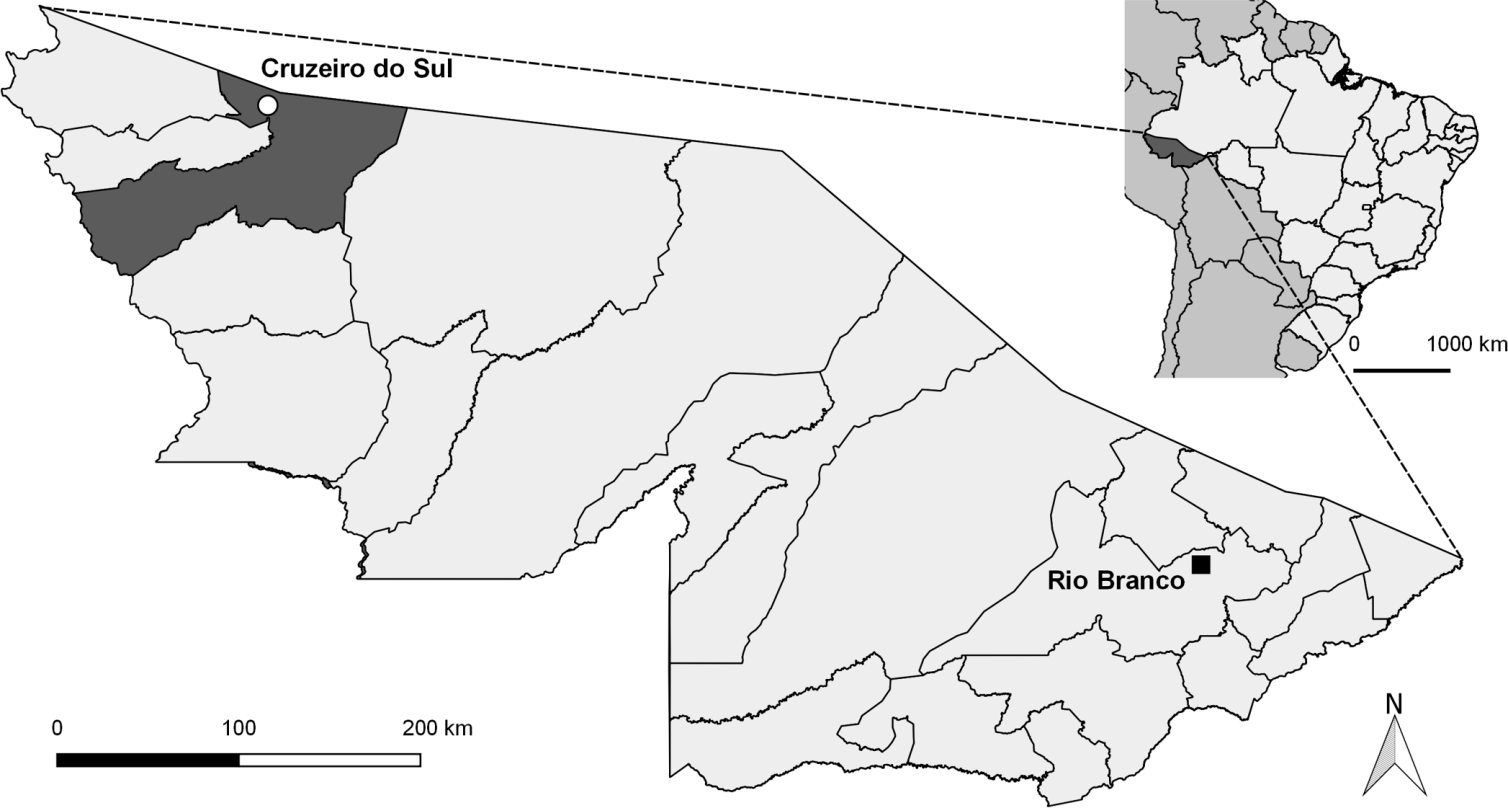
Map showing the geographic location of Cruzeiro do Sul, Acre State, Brazilian Amazon. Cruzeiro do Sul has an estimated population of 82,075 inhabitants. The map also indicates Rio Branco, the capital of Acre state.

This is a population-based observational study developed in the city of Cruzeiro do Sul—Acre (Brazil), located in the Brazilian Amazonian region (7°37’51”S, 72°40’12”W) (Fig 1). Cruzeiro do Sul has an estimated population of 82,075 inhabitants and an average of 1,650 births per year [20]. Together with Porto Velho and Manaus, the three cities are responsible for 21.9% of the malaria cases notified in Brazil [21]. It is a high transmission risk city, with an annual parasitic incidence of 214 cases per 1,000 inhabitants, with the prevalence of *P*. *vivax* infection [21]. The universe of the studied population was composed of all live newborns delivered by women living in the city, between January 2006 and December 2014. The information regarding the mother, newborn and delivery was extracted from SINASC, and the information on the malaria episodes and parasite species was obtained from the SIVEP-Malaria. By knowing that the primary health care is provided free of cost in Brazil and that the information systems of the Ministry of Health (MoH) offer wide coverage, we can presume that these datasets are reliable.

### Ethical considerations

The consent to use this dataset was granted by the Secretaries of Health from the State of Acre and from the city of Cruzeiro do Sul after signing the Term of Commitment for the Use of Data from Medical Records under the agreement of maintaining confidentiality and safety of the collected data from the medical records and databases. The nominal data was strictly used for the record linkage, being the deidentification done before data analysis. In accordance, this study is in agreement with the Resolution nº 196/96 of the Brazilian National Health Committee. Ethical clearance was provided by the Committees for Research of the University of São Paulo and the Federal University of Acre (Plataforma Brasil, CAAE: 03930812.8.0000.5467 and 03930812.8.3001.5010, respectively).

### Exclusion criteria

**Fig 2:**
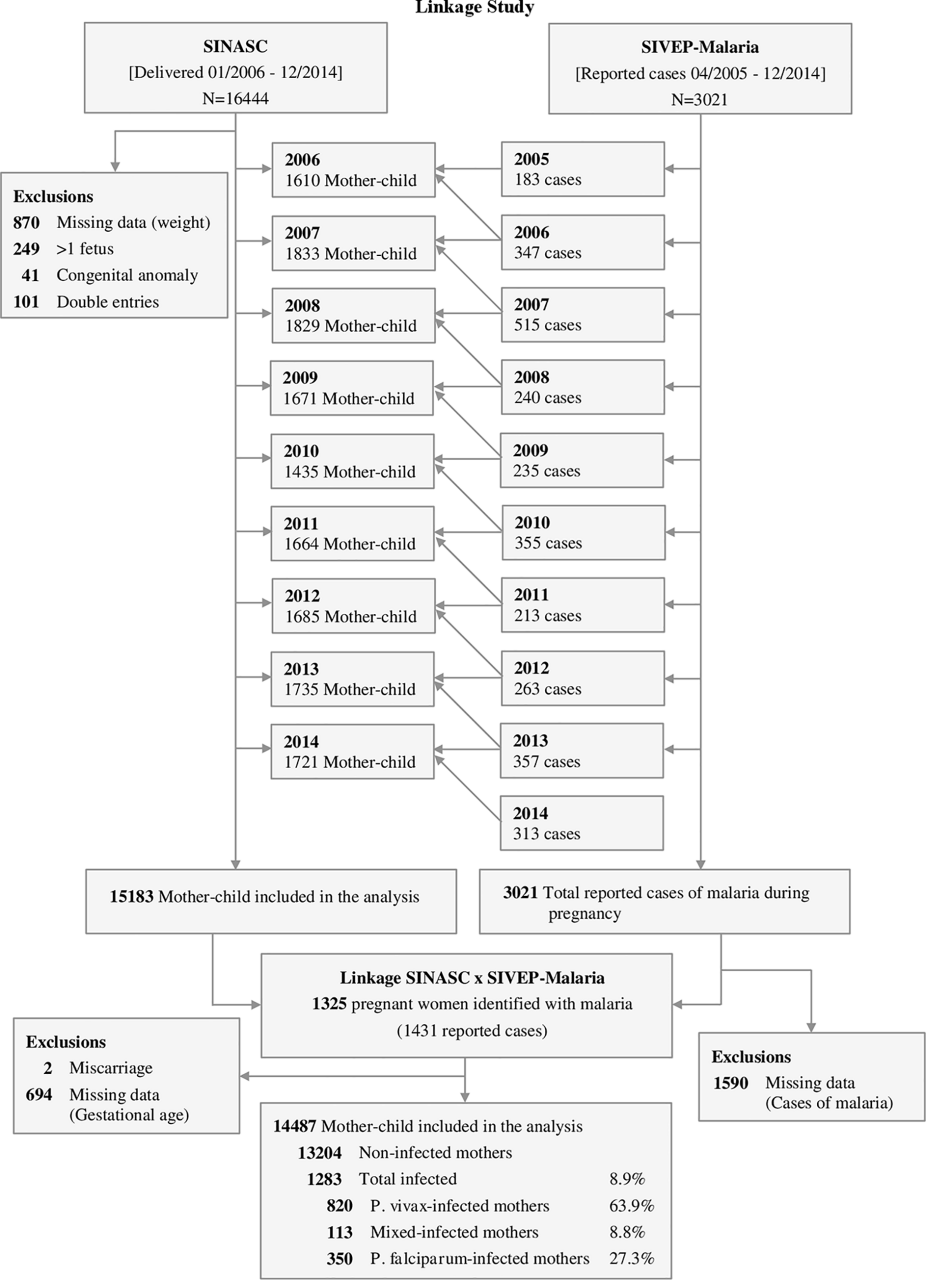
Flowchart detailing exclusion criteria applied to the evaluation of the enrolled maternal-child pairs. *P*. *vivax infected mothers—one or more infections only by P*. *vivax; P*. *falciparum infected mothers—one or more infections only by P*. *falciparum;* Mixed infection—*P*. *vivax*- and *P*. *falciparum*-infection occurring at the same time and/or at different times during pregnancy.

In this study, the SINASC database was considered the reference. Before performing the record linkage, curation was performed, and newborns with double entries, lack of information on birth weight, presenting congenital diseases or twins were excluded. Upon the linkage of SINASC with SIVEP-Malaria database, newborns with less than 22 weeks’ gestational age (miscarriage) or with no information on the gestational age at birth were excluded (Fig 2).

### Screening of malaria infection

In Brazil, whenever individual show suspicious malaria symptoms, it is tested by qualified endemic agents that monitor micro-regions. The gold standard method for malaria diagnosis is the thin and thick blood smear, which is screened by trained microscopists from the System of Epidemiological Surveillance of the MoH, and further revised by senior experts, to confirm the results. Infections were categorized per parasite species: *P*. *falciparum*, *P*. *vivax*, or mixed infections. All women who had malaria during pregnancy were treated with antimalarial drugs under medical prescription, according to the Brazilian MoH guidelines.

### Definitions and gestational age estimation

LBW was defined as birth weight < 2500 grams (g). WHO child growth standards were used to classify the small for gestational age newborns, weight ≤ 10th centile (boys ≤ 2758 g, girls ≤ 2678g). The very preterm birth was defined as birth between ≥28 and <32 weeks’ gestation; late preterm birth was defined as birth between ≥32 and <37 weeks’ gestation, and total preterm birth was defined as birth <37 weeks’ gestation [22]. The gestational age was established by the woman’s last menstrual period and, when possible, adjusted by ultrasound during antenatal visit care. In SINASC database, gestational age is categorized as follows: less than 22 weeks’ gestation, 22–27 weeks, 28–31 weeks, 32–36 weeks, 37–41 weeks, and 42 weeks or more.

### Record linkage strategy

The record linkage was performed by using the RecLink III software [23] through the deterministic method (manual search). For the data preprocessing, standardization of both databases was performed by withdrawing accentuations, extra spaces, special characters, and prepositions. After, databases were unified only by two shared variables that presented the appropriate fulfillment. Each year of the record linkage corresponds to one year of the SINASC (containing births records) assembled with two years of the SIVEP-Malaria, to identify all malaria cases presented by women during pregnancy. The linked database gathered the variables from SINASC (mother age, gestational age at delivery, gravidity, number of antenatal visits, birth weight, and type of birth), with variables from SIVEP-Malaria (infection by *Plasmodium* spp. (yes / no) and parasite species).

## Statistical analysis

Data were extracted into Microsoft Excel, and Stata 14.2 and GraphPad Prism software were used for statistical analyses. We used descriptive statistics to assess the distribution of all continuous (means and standard deviation [SD] or median and interquartile ranges [IQR]), and categorical (frequencies and percentages) variables. Differences between groups were evaluated using Mann-Whitney U-tests, accordingly. Categorical data and proportions were analyzed using chi-square tests. Every p values were 2-sided at a significance level of <0.05. To assess the association between malaria and birth weight reduction or prematurity, adjusted odds ratios (OR) with 95% confidence intervals (CI) were estimated using a multivariate logistic regression approach. These models included infections by malaria (no / yes), parasite species (no / *P*. *vivax*, *P*. *falciparum and mixed* infections), maternal age (≥ 18 years old / ≤ 17 years old), gravidity (primigravida / multigravida), and years of formal education (≥ 4 years / ≤ 3 years) as explanatory variables, and birth weight [≤ 10th centile] (yes / no) or LBW (yes / no) as response variables. The first category for each explanatory variable was considered as reference [24].

## Results

### Study population and baseline characteristics

**Fig 3:**
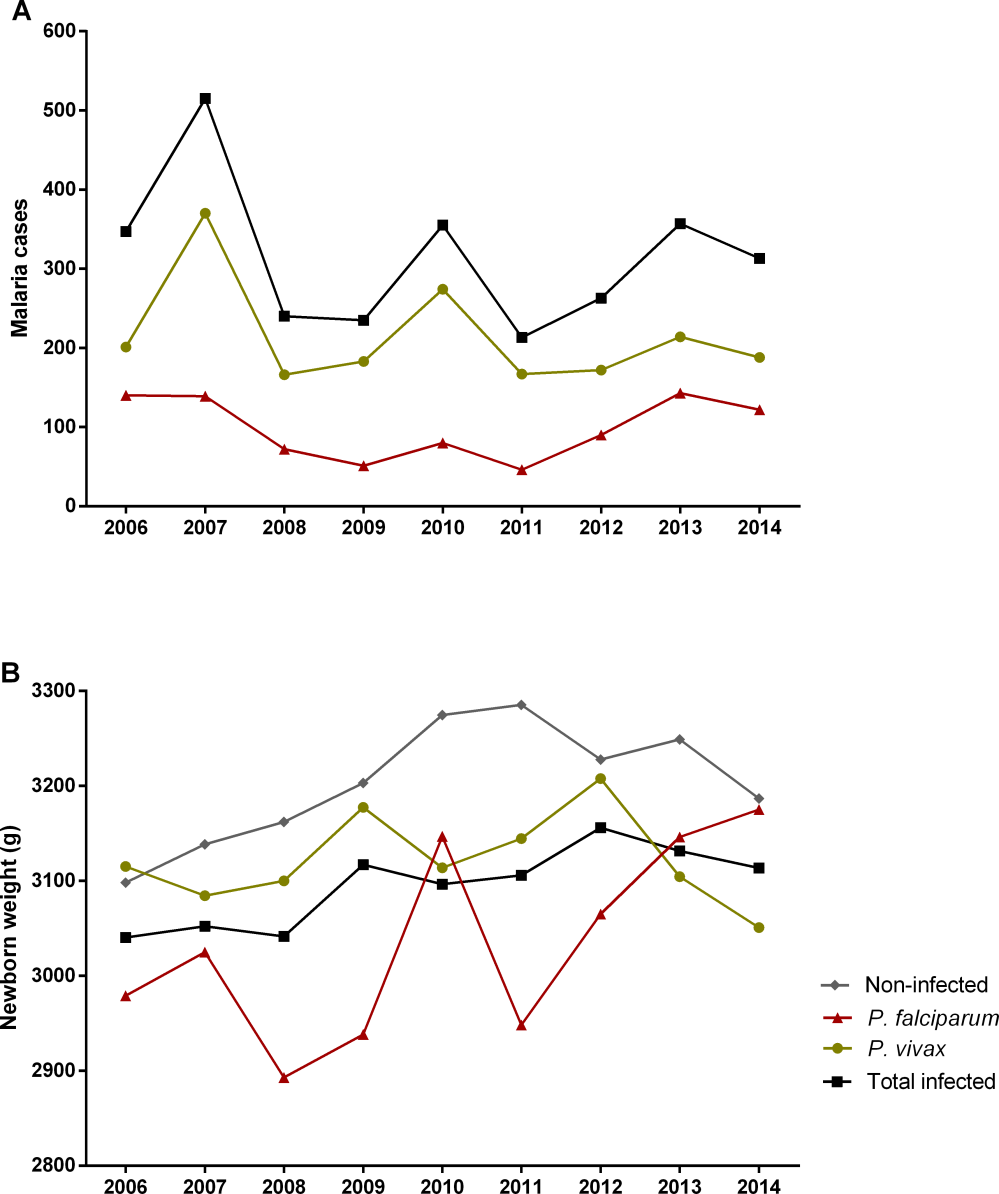
Time-series of gestational malaria cases between 2006–2014. (A) Number of gestational malaria cases per species, (B) mean birth weight of newborns from non-infected and infected women during pregnancy.

Between January 2006 and December 2014, 16,444 live births occurred in Cruzeiro do Sul (Acre) with a total of 3,021 malaria cases notified during pregnancy. At the end of the linkage process, 1431 (47.4%) cases of malaria were identified in pregnant women. After applying the exclusion criteria, 14,487 (88.1%) maternal-child pairs remained for further analysis (Fig 2). Table 1 shows maternal characteristics according to infection status (detailed by year in the S1 Table). To highlight that: circa 35% of women were primigravida; above 40% had at least 8 years of formal education (despite the high proportion of no-schooling women); and more than 70% had a minimum of four antenatal visits (Table 1). Nevertheless, it was possible to observe that there were no major differences between non-infected and infected mothers. Malaria incidence in the studied population was 8.9%, with *P*. *vivax* contributing to 63.9% of the cases (Fig 2). Time series of malaria cases in pregnant women allowed to detect three epidemic peaks along the studied period, one in 2007 with more than 500 cases, and other two in 2010 and 2013 (Fig 3A and S2 Table). Interestingly, the significant reduction of cases from 2007 to 2008 coincides with the introduction of artemisinin combined therapy in Brazil [17]. Though, *P*. *falciparum* infections represented on average more than 30% of cases reported during pregnancy, in the assessed years (S2 Table).

**Table 1:** Baseline characteristics of mothers at delivery.

Characteristics	Non-infected (N = 13,204)	Malaria ^a^ (N = 1,283)	*P*. *vivax* (N = 820)	*P*. *falciparum* (N = 350)	Mixed (N = 113)
Maternal age (years), mean (SD) ^b^	24.3 ± 6.4	23.3 ± 6.0	23.3 ± 5.9	24.0 ± 6.3	21.9 ± 5.8
Primigravida, no. (%) ^c^	4798 (36.3)	439 (34.2)	299 (36.5)	98 (28.0)	42 (37.2)
Male newborns, no. (%)	6860 (52.0)	675 (52.6)	425 (51.8)	189 (54.0)	61 (54.0)
Gestational age, no. (%)					
22–27 weeks	15 (0.1)	3 (0.2)	3 (0.4)	0	0
28–31 weeks	69 (0.5)	12 (0.9)	9 (1.1)	2 (0.6)	1 (0.9)
32–36 weeks	884 (6.7)	97 (7.6)	52 (6.3)	35 (10.0)	10 (8.9)
37 weeks or more	12236 (92.7)	1171 (91.3)	756 (92.2)	313 (89.4)	102 (90.2)
Years of formal education, no. (%) ^d^^,^^f^					
No formal education	1894 (14.5)	215 (16.9)	117 (14.4)	75 (21.7)	23 (20.5)
1–3 years	1204 (9.2)	120 (9.5)	73 (9.0)	35 (10.1)	12 (10.7)
4–7 years	2866 (22.0)	338 (26.6)	219 (27.0)	90 (26.1)	29 (25.9)
8–11 years	5041 (38.6)	467 (36.8)	308 (37.9)	122 (35.4)	37 (33.0)
12 or more	1990 (15.2)	122 (9.6)	91 (11.2)	21 (6.1)	10 (8.9)
Antenatal care visit, mean (SD) ^e^^,^^f^					
None	1295 (9.9)	86 (6.7)	39 (4.8)	39 (11.1)	8 (7.1)
1–3 visits	2100 (16.0)	211 (16.5)	130 (16.0)	65 (18.6)	16 (14.3)
4–6 visits	4004 (30.5)	417 (32.7)	281 (34.5)	109 (31.1)	27 (24.1)
7 or more	5676 (43.2)	560 (43.9)	363 (44.6)	136 (38.9)	61 (54.5)

N, number of individuals; SD, standard deviation; no., number of events.

^a^ Malaria group consists of total pregnant women who had an infection (*P*. *falciparum* and *P*. *vivax* or both).

^b^ Maternal age was recorded in 13,202 non-infected pregnant women.

^c^ Information generated based on the number of live births and deaths, reported by the mother.

^d^ Years of formal education were recorded in 13,054 non-infected and 1,269 infected pregnant women (812 *P*. *vivax* and 345 *P*. *falciparum*).

^e^ Antenatal visits were recorded in 13,138 non-infected and 1,276 infected pregnant women (814 *P*. *vivax*).

^f^ Theses variables have ignored values.

### Association of MiP with reduction of the newborn birth weight

The analysis of the newborn birth weight across the nine years period, allowed to observe a significant reduction in the mean weight of babies born from women that had malaria during pregnancy (Fig 3B, Table 2, and S3 Table). Newborns from *P*. *falciparum*-infected mothers presented a more prominent difference of approximately 150 g (p<0.0001) when compared to newborns from non-infected women (Table 2). Notably, the comparison of each group by year evidenced that newborns from *P*. *vivax*-infected mothers showed higher weight reduction when compared with non-infected (S3 Table). These differences can be explained by the higher prevalence of newborns with LBW among *P*. *vivax*-infected women (term LBW: NI 4.8%, Pv 6.5%, p = 0.031; all LBW: NI 6.8%, Pv 8.9%, p = 0.020) (Table 2 and S4 Table). Although this prevalence occurred throughout the assessed years, it was more evident in 2006 and 2013 (S4 Table).

**Table 2:** Clinical outcomes of newborns at birth.

Characteristics	Non-infected (N = 13,204)	Malaria ^a^ (N = 1,283)	p value ^b^	*P*. *vivax* (N = 820)	p value ^b^	*P*. *falciparum* (N = 350)	p value ^b^	Mixed (N = 113)	p value ^b^
All birth weight (g)			<0.0001		<0.0001		<0.0001		0.0002
Mean (SD)	3200 ± 514.7	3090 ± 524.2		3119 ± 532.4		3050 ± 506.7		3006 ± 503.9	
Median (IQR)	3215 (2900–3530)	3100 (2785–3420)		3133 (2800–3448)		3030 (2780–3350)		3095 (2690–3330)	
Term birth weight (g) ^c^			<0.0001		<0.0001		<0.0001		0.004
Mean (SD)	3236 ± 482.2	3140 ± 471.6		3160 ± 486.0		3106 ± 447.9		3094 ± 426.9	
Median (IQR)	3240 (2940–3550)	3130 (2840–3430)		3150 (2843–3453)		3060 (2850–3380)		3155 (2770–3348)	
All low birth weight, no. (%)	896 (6.8)	120 (9.4)	0.001	73 (8.9)	0.020	31 (8.9)	0.130	16 (14.2)	0.002
Term low birth weight, no. (%) ^c^	581 (4.8)	74 (6.3)	0.017	49 (6.5)	0.031	17 (5.4)	0.575	8 (7.8)	0.144
Prematurity, no. (%) ^d^	968 (7.3)	112 (8.7)	0.069	64 (7.8)	0.614	37 (10.6)	0.022	11 (9.7)	0.330
Very preterm birth, no. (%) ^e^	69 (7.1)	12 (10.7)	0.058	9 (14.1)	0.032	2 (5.4)	0.901	1 (9.1)	0.596
Late preterm birth, no. (%) ^f^	884 (91.3)	97 (86.6)	0.239	52 (81.3)	0.694	35 (94.6)	0.015	10 (90.9)	0.362
Very preterm birth weight (g)			0.868		0.863		0.627		0.638
Mean (SD)	2036 ± 722.2	2032 ± 625.5		1979 ± 692.0		2113 ± 576.3		2353	
Median (IQR)	1880 (1460–2575)	2045 (1533–2548)		1915 (1360–2575)		2113 (1705–2520)		2353	
Late preterm birth weight (g)			0.093		0.838		0.164		0.0009
Mean (SD)	2814 ± 621.6	2683 ± 677.2		2839 ± 645.6		2599 ± 703.5		2168 ± 444.1	
Median (IQR)	2840 (2423–3233)	2720 (2240–3100)		2798 (2398–3268)		2765 (2320–3075)		2225 (1860–2500)	

N, number of individuals; no., number of events; SD, standard deviation; IQR, interquartile range; g, grams.

^a^ Malaria group consists of total pregnant women who had an infection (*P*. *falciparum* and *P*. *vivax* or both).

^b^ Statistical tests were applied according to the type of variable (Mann-Whitney or Chi-square), and compare Malaria, *P*. *vivax*, *P*. *falciparum* and Mixed infected groups versus Non-infected.

^c^ Term indicates all babies born at 37 weeks’ gestation or later.

^d^ Prematurity was defined as birth <37 weeks’ gestation.

^e^ Very preterm birth was defined as birth between ≥28 and <32 weeks’ gestation.

^f^ Late preterm birth was defined as birth between ≥32 and <37 weeks’ gestation.

**Fig 4:**
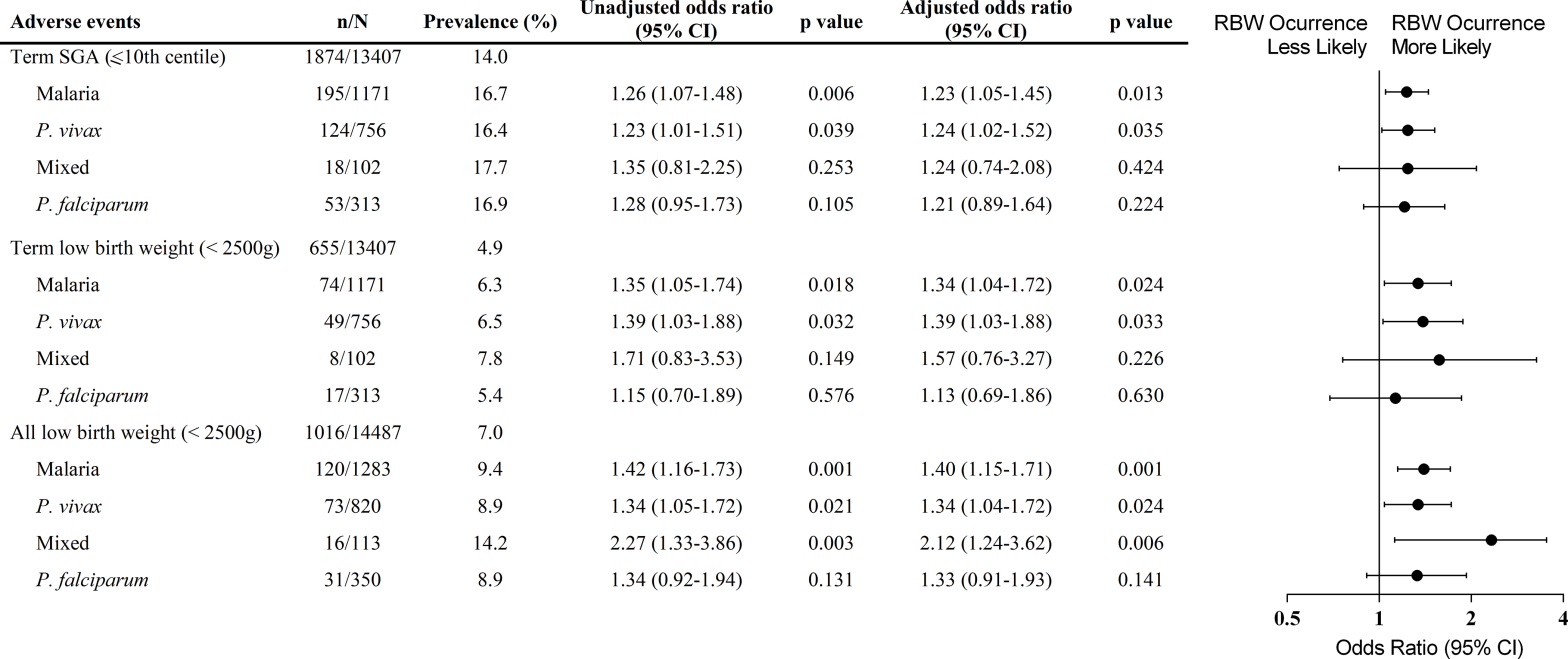
Forest plot of the odds ratio for weight reduction in newborns from women infected during pregnancy compared to babies from non-infected women, according to *Plasmodium* species. Each model adjusting for maternal age, gravidity and years of formal education (less than 4 years); mixed infection (*P*. *vivax* and *P*. *falciparum*-infection). p values were estimated through logistic regression methods. n, number of events; N, total number in each group; CI, confidence interval; SGA, small for gestational age; LBW, low birth weight.

**Fig 5:**
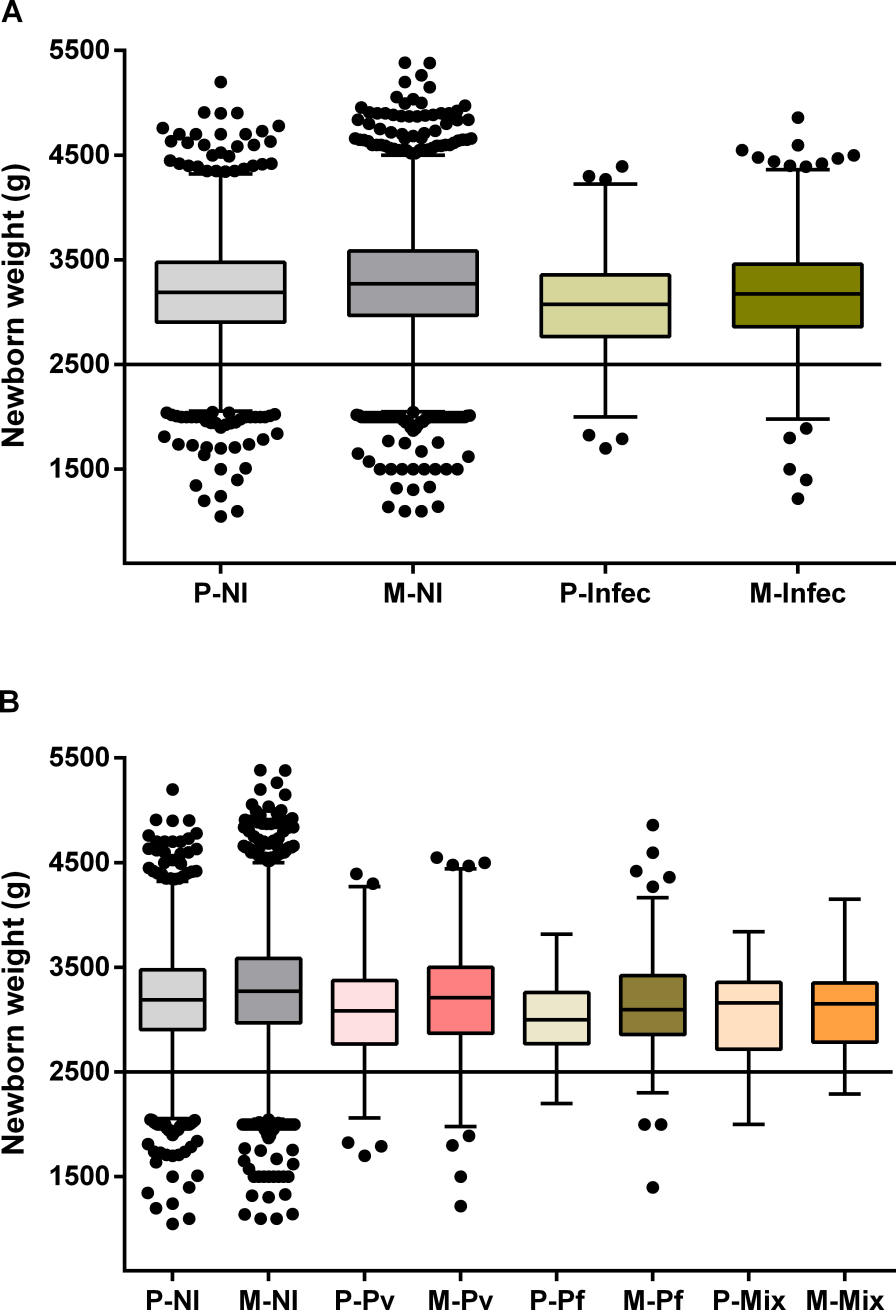
Impact of malaria on birth weight at term according to gravidity. Tukey boxplots show the effect of gravidity on the weight of newborns from malaria-infected women (A), and on newborns from women infected according with *Plasmodium* species (B). The bottom and the top of the box are the first and third quartiles, the line inside the box is the median, and the whiskers represent the lowest and the highest data within 1.5 IQR of the first and upper quartiles. The line indicates the cut-off of low birth weight. Differences between each group were examined with Mann-Whitney or Kruskal-Wallis test with a Dunn’s post hoc test. (A) P—NI x Infec (p<0.0001); M—NI x Infec (p<0.0001); NI- P x M (p<0.0001); and Infec—P x M (p = 0.0004). (B) P—NI x Pv (p = 0.0001); NI x Pf (p = 0.0003); M—NI x Pv (p = 0.0009); NI x Pf (p<0.0001); NI x Mix (p = 0.003); Pv x Pf (p = 0.025); Pv—P x M (p = 0.0009). P, primigravida; M, multigravida; NI, non-infected pregnant women; Infec, infected pregnant women; Pv, *P*. *vivax*-infection; Pf, *P*. *falciparum*-infection; Mix, mixed-infection.

Using multivariate logistic regression analysis malaria was associated with an increased odds of newborn small for gestational age (SGA) at term (weight ≤ 10th centile, boys ≤ 2758g and girls ≤ 2658g) (Odds ratio [OR] 1.23, 95%, confidence interval [CI] 1.05–1.45, p = 0.013), which relates to *P*. *vivax* infection (OR 1.24, 95% CI 1.02–1.52, p = 0.035) (Fig 4). Moreover, LBW at term was significantly increased in newborns from malaria-infected mothers, (OR 1.34, 95% CI 1.04–1.72, p = 0.024), which was evidenced when mothers were infected by *P*. *vivax* (OR 1.39, 95% CI 1.03–1.88, p = 0.033) (Fig 4). Additionally, segregation by gravidity showed that newborns at term from both primigravida and multigravida presented reduced birth weight when mothers had malaria during pregnancy, irrespective of species (Fig 5 and S5 Table). Nevertheless, newborns from primigravida showed a more prominent birth weight reduction upon infection (Fig 5B and S5 Table).

### *P*. *falciparum* infection during pregnancy increases preterm births

**Fig 6:**
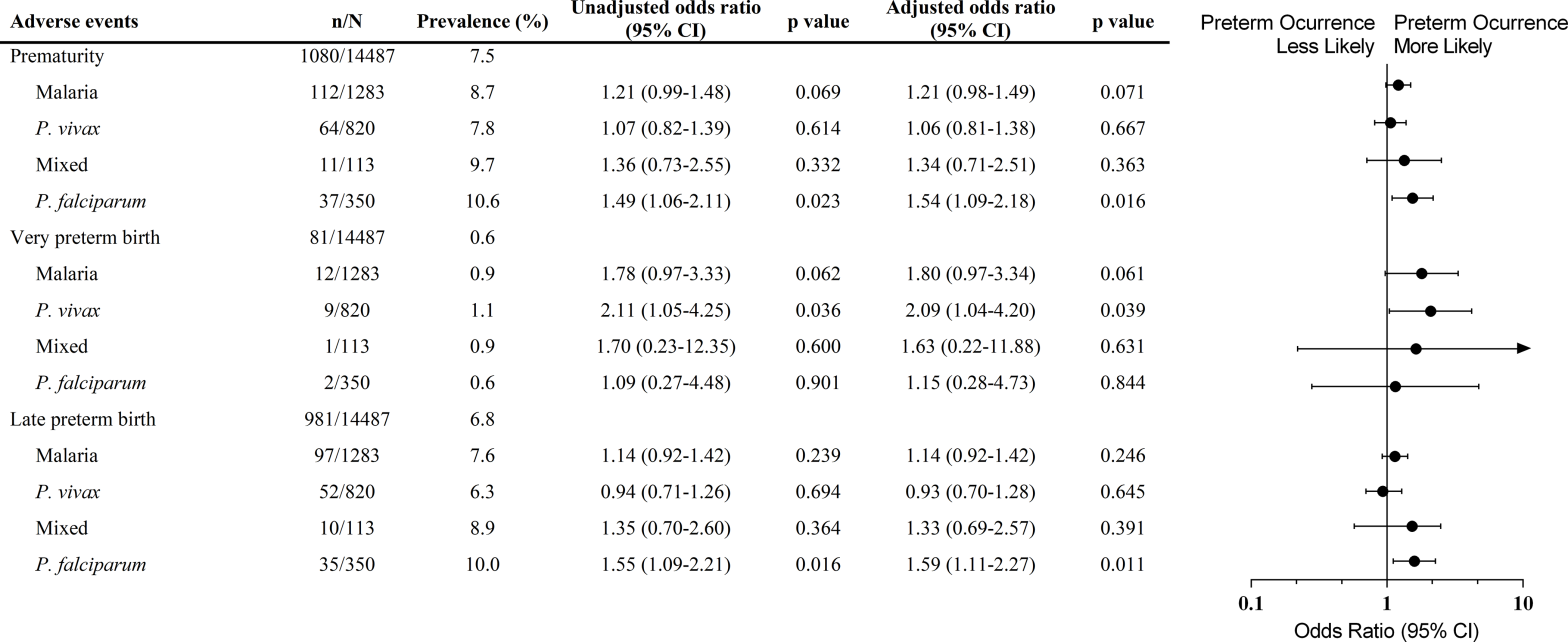
Forest plot of the odds ratio for prematurity in newborns from women infected during pregnancy compared to babies from non-infected women, according to *Plasmodium* species. Each model adjusting for maternal age, gravidity and years of formal education (less than 4 years). Mixed infection (*P*. *vivax* and *P*. *falciparum*-infection). p values were estimated through logistic regression methods. n, number of events; N, total number in each group; CI, confidence interval. Prematurity was defined as birth <37 weeks’ gestation; very preterm birth was defined as birth between ≥28 and <32 weeks’ gestation, and late preterm birth was defined as birth between ≥32 and <37 weeks’ gestation.

The assembly of databases unveiled increased prematurity among babies born from *P*. *falciparum*-infected women during pregnancy (Table 2). Prematurity prevalence increased around 3% when women were infected with *P*. *falciparum*, and the association was evidenced by multivariate logistic regression analysis (OR 1.54, 95% CI 1.09–2.18, p = 0.016), which corresponded with late preterm births (OR 1.59, 95% CI 1.11–2.27, p = 0.011) (Fig 6). Moreover, *P*. *vivax* infections were related to very preterm births in women with malaria during pregnancy (OR 2.09, 95% CI 1.04–4.20, p = 0.039) (Fig 6).

Together, these results demonstrate that linkage of national record databases is a valuable research tool, which disclosed adverse neonatal outcomes upon malaria infection during pregnancy in Brazil.

## Discussion

Malaria during pregnancy is known as an important risk factor for miscarriage, stillbirth, LBW and maternal anemia [9,25–27]. Nevertheless, little is known about MiP in the Americas endemic regions, where it predominates *P*. *vivax* infections. This work is the first to assess the effect of MiP in Brazil through the linkage of national databases of the Brazilian Ministry of Health, SINASC and SIVEP-Malaria. Interestingly, despite the decrease in the number of MiP cases through the studied period (2006–2014), the impact of malaria during pregnancy is still evident. The reduction of the mean of the weight was maintained throughout the years and the higher prevalence of preterm births among newborns from women that presented malaria during pregnancy.

The number of studies estimating the real frequency of MiP women is still limited, both in Brazil and in other regions of the Americas, which are considered low transmission areas [3]. The prevalence (8.9%) of malaria during pregnancy in our study was similar to the findings of a multi-centric study that enrolled data from the Americas (Guatemala, Colombia, and Brazil) between 2008–2011, and another study performed in Urubá (Colombia) between 2005–2009 [28,29]. Though, the prevalence is higher in relation to reports from Iquitos (6.6%) (Peru), and from other Brazilian cities, such as Manaus (6.1%) and Coari (4.3%) in the Amazonas state, and Rio Branco (1.4%) in Acre state [30–33]. The discrepancies may encompass differential study designs and endemicity of studied areas.

Prematurity is one of the adverse effects commonly observed in malaria during pregnancy [7,34,35]. Usually, it correlates with infections occurring during the third trimester of pregnancy and contributes to increasing the number of newborns with LBW, which is more likely to be observed in low transmission areas [3,36]. In fact, our data show that *P*. *falciparum* infections during pregnancy are responsible for a high proportion of preterm births, mainly late preterm births (≥32 and <37 weeks of gestation). However, it was not possible to correlate the time of infection with the gestational trimester.

Newborns reduced weight at birth either classified as LBW or SGA, is an important predictive marker of neonatal and child survival, and can result from two basic factors: intrauterine growth restriction and preterm births [35,37]. In MiP, birth weight reduction is the main adverse outcome observed in studies involving *P*. *falciparum* infections [3,34,38,39]. In our observations, malaria infection during pregnancy represents a critical morbidity that impacts newborns’ weight. The records show that malaria in pregnant women increases the number of babies born at term with SGA by 19.3% and LBW by 28.6% (SGA–NI 14.0%, Malaria 16.7%; term LBW–NI 4.9%, Malaria 6.3%). Strikingly, *P*. *vivax* infection during pregnancy represents the higher odds for the occurrence of birth weight reduction (SGA—OR 1.24, 95% CI 1.02–1.52, p = 0.035; term LBW—OR 1.39, 95% CI 1.03–1.88, p = 0.033). The absence of the association between *P*. *falciparum* infections and the occurrence of SGA or LBW newborns can be related with the restricted pool of variant genes of the Amazonian parasite, which can explain the mild outcomes observed in the Americas, substantially different from other endemic regions in the world [40]. Nevertheless, our data corroborate some findings in Southeast Asia from Moore *et al*. that show that *P*. *vivax* infection is associated with SGA and *P*. *falciparum* infection with late preterm, although we could not correlate with time of infection [38].

The reduction of newborns birth weight is multifactorial, and it can be related to social-economic, environmental, nutritional, and clinic factors during pregnancy. However, in this study, it was not possible to assess other risk factors, once these variables were absent in the databases used. Of note, it is important to highlight that it is impossible to compare this study with other carried out in Africa. There, *P*. *falciparum* infections are predominant and, in general, the health systems that diagnose and treat malaria have several limitations, summed up with the high rate of co-infection with other diseases, such as HIV and tuberculosis. The Brazilian Amazonian region has a health care system with effective strategies to control, diagnose, and treat malaria, despite being a low transmission area with predominance of *P*. *vivax* infections. These characteristics make our findings even more interesting, as we observed a substantial impact of infection during pregnancy in newborns.

In Brazil, malaria is a mandatory notification disease, and SIVEP-Malaria is essential to plan health interventions that enable effective control and preventive strategies to eradicate the disease. For pregnant women, the early diagnosis is essential to prevent adverse outcomes. In 2014, it was enforced, by Brazilian MoH, a malaria routine screen during antenatal care and at delivery, in women living in Brazilian Amazonian region states [41]. This initiative brought important benefits for both mother and fetus, enabling early treatment and preventing gestational adverse outcomes.

This work present potential limitations. First, the databases used have only two shared variables with adequate fulfillment, and this hampered the identification of all women with about 52.6% of cases not matched. Therefore, the number of pregnant women with malaria can be underestimated. Second, the reduction of birth weight has different etiologies. Although we used important exclusion criteria, it was not possible to identify through SINASC women presenting other infections, such as TORCHs, as well as, nutritional or other risk factors.

## Conclusions

In conclusion, this work allowed us to observe through a population-based observational study, the effect of MiP on newborns birth weight in a region considered of low transmission and with *P*. *vivax* infections predominance. During the evaluated period (2006–2014), malaria infections continue to be an important risk factor for prematurity and reduction of newborns’ birth weight, despite the decline in the number of cases reported in the region. We have shown that the SINASC and the SIVEP-Malaria databases linkage allow to estimate the extent of malaria adverse effects, which permit to improve information and further plan interventions. These findings reinforce the urgent need for health programs and actions to prevent and protect pregnant women against the consequences of malaria, especially during the antenatal care.

## Supporting information

S1 Table Characteristics of mothers and newborns per year in Cruzeiro do Sul, 2006–2014. Data from the System Information of Live Births provided by the Cruzeiro do Sul Municipal Secretariat of Health. ^a^ Information generated based on the number of live births and deaths reported by the mother. ^b^ There are other groups with ignored values.
(DOCX)

S2 Table Trends in malaria infection during pregnancy in Cruzeiro do Sul, 2006–2014. N, number of malaria cases. Data are N or N (%). Values correspond to the total number of malaria episodes reported between 2006 and 2014.
(DOCX)

S3 Table Description of the birth weight of newborns from Non-Infected and Infected pregnant women per year. N, number of individuals; IQR, interquartile range. ^a^ Malaria group consists of total pregnant women who had an infection (*P*. *falciparum*, *P*. *vivax*, and Mixed infections). ^b^ Differences between Non-Infected and the other groups were evaluated using Mann-Whitney rank sum tests.
(DOCX)

S4 Table Description of term low birth weight newborns from Non-Infected and Infected pregnant women per year. N, number of individuals; no., number of newborns with low birth weight. ^a^ Malaria group consists of total pregnant women who had an infection (*P*. *falciparum*, *P*. *vivax*, and Mixed infections). ^b^ Differences between Non-Infected and Infected groups were evaluated using Chi-square tests.
(DOCX)

S5 Table Description of birth weight of term newborns from Non-Infected and Infected pregnant women per gravidity. N, number of individuals; SD, standard deviation; IQR, interquartile range. ^a^ Malaria group consists of total pregnant women who had an infection (*P*. *falciparum*, *P*. *vivax*, and Mixed infections). ^b^ Differences between each group were examined using Mann-Whitney or Kruskal-Wallis test with Dunn post hoc test. ^c^ p = 0.025 for *P*. *vivax* versus *P*. *falciparum* groups.
(DOCX)

## References

[1] Rogerson SJ, Desai M, Mayor A, Sicuri E, Taylor SM, van Eijk AM. Burden, pathology, and costs of malaria in pregnancy: new developments for an old problem. Lancet Infect Dis. 2018; doi: 10.1016/S1473-3099(18)30066-5 10.1016/S1473-3099(18)30066-5 29396010

[2] Dellicour S, Tatem AJ, Guerra CA, Snow RW, Ter Kuile FO. Quantifying the number of pregnancies at risk of malaria in 2007: A demographic study. PLoS Med. 2010;7: 1–10. doi: 10.1371/journal.pmed.1000221 20126256 10.1371/journal.pmed.1000221 PMC2811150

[3] Desai M, ter Kuile FO, Nosten F, McGready R, Asamoa K, Brabin B, et al Epidemiology and burden of malaria in pregnancy. Lancet Infect Dis. 2007;7: 93–104. doi: 10.1016/S1473-3099(07)70021-X 17251080 10.1016/S1473-3099(07)70021-X

[4] Lagerberg RE. Malaria in pregnancy: a literature review. J Midwifery Womens Health. 2008;53: 209–15. doi: 10.1016/j.jmwh.2008.02.012 18455095 10.1016/j.jmwh.2008.02.012

[5] Rogerson SJ, Hviid L, Duffy PE, Leke RF, Taylor DW. Malaria in pregnancy: pathogenesis and immunity. Lancet Infect Dis. Elsevier; 2007;7: 105–117. doi: 10.1016/S1473-3099(07)70022-1 10.1016/S1473-3099(07)70022-1 17251081

[6] Lawn JE, Blencowe H, Oza S, You D, Lee AC, Waiswa P, et al Every Newborn: progress, priorities, and potential beyond survival. Lancet. 2014;384: 189–205. doi: 10.1016/S0140-6736(14)60496-7 24853593 10.1016/S0140-6736(14)60496-7

[7] Umbers AJ, Aitken EH, Rogerson SJ. Malaria in pregnancy: small babies, big problem. Trends Parasitol. Elsevier Ltd; 2011;27: 168–75. doi: 10.1016/j.pt.2011.01.007 21377424 10.1016/j.pt.2011.01.007

[8] Steketee RW, Wirima JJ, Hightower AW, Slutsker L, Heymann DL, Breman JG. The effect of malaria and malaria prevention in pregnancy on offspring birthweight, prematurity, and intrauterine growth retardation in rural Malawi. Am J Trop Med Hyg. 1996;55: 33–41. Available: http://www.ncbi.nlm.nih.gov/pubmed/8702035 8702035 10.4269/ajtmh.1996.55.33

[9] Guyatt HL, Snow RW. Impact of Malaria during Pregnancy on Low Birth Weight in Sub-Saharan Africa. Clin Microbiol Rev. 2004;17: 760–769. doi: 10.1128/CMR.17.4.760-769.2004 15489346 10.1128/CMR.17.4.760-769.2004 PMC523568

[10] Nosten F, McGready R, Simpson JA, Thwai KL, Balkan S, Cho T, et al Effects of Plasmodium vivax malaria in pregnancy. Lancet. 1999;354: 546–549. doi: 10.1016/S0140-6736(98)09247-2 10470698 10.1016/s0140-6736(98)09247-2

[11] McGready R, Lee SJ, Wiladphaingern J, Ashley E a., Rijken MJ, Boel M, et al Adverse effects of falciparum and vivax malaria and the safety of antimalarial treatment in early pregnancy: A population-based study. Lancet Infect Dis. 2012;12: 388–396. doi: 10.1016/S1473-3099(11)70339-5 22169409 10.1016/S1473-3099(11)70339-5 PMC3346948

[12] Moore KA, Fowkes FJI, Wiladphaingern J, Wai NS, Paw MK, Pimanpanarak M, et al Mediation of the effect of malaria in pregnancy on stillbirth and neonatal death in an area of low transmission: observational data analysis. BMC Med. 2017;15: 98 doi: 10.1186/s12916-017-0863-z 28486979 10.1186/s12916-017-0863-z PMC5424335

[13] World Health Organization. World Malaria Report 2016 [Internet]. Geneva; 2016 Nov. Available: http://www.who.int/malaria/publications/world-malaria-report-2016/report/en/

[14] Guimarães EA de A, Loyola Filho AI de, Hartz ZM de A, de Meira AJ, Luz ZMP. A descentralização do SINASC e a completitude das variáveis da declaração de nascido vivo em municípios mineiros de 1998 a 2005. Rev Bras Crescimento Desenvolv Hum. 2011;21: 832–840.

[15] Paiva NS, Coeli CM, Moreno AB, Guimarães RM, Camargo Júnior KR. Sistema de informações sobre nascidos vivos: um estudo de revisão. Ciência e Saúde Coletiva. 2011;16: 1211–1220. 21503469 10.1590/s1413-81232011000700053

[16] Romero DE, da Cunha CB. Avaliação da qualidade das variáveis epidemiológicas e demográficas do Sistema de Informações sobre Nascidos Vivos, 2002. Cad Saude Publica. 2007;23: 701–714. doi: 10.1590/S0102-311X2007000300028 17334583 10.1590/s0102-311x2007000300028

[17] Oliveira-Ferreira J, Lacerda MV, Brasil P, Ladislau JL, Tauil PL, Daniel-Ribeiro CT. Malaria in Brazil: an overview. Malar J. 2010;9: 115 doi: 10.1186/1475-2875-9-115 20433744 10.1186/1475-2875-9-115 PMC2891813

[18] Brazilian Ministry of Health, Fundação Nacional da Saúde. Manual de Procedimento do Sistema de Informações sobre Mortalidade [Internet]. Brasília; 2001. Available: http://bvsms.saude.gov.br/bvs/publicacoes/sis_mortalidade.pdf

[19] Jorge MHP de M, Gawryszewski VP, Latorre M do RD de O. I—Análise dos dados de mortalidade. Rev Saude Publica. 1997;31: 05–25. doi: 10.1590/S0034-89101997000500002 9595755

[20] Instituto Brasileiro de Geografia e Estatística. Contagem da População [Internet]. 2010. Available: http://www.censo2010.ibge.gov.br/resultados_do_censo2010.php

[21] Brazilian Ministry of Health, Secretaria de Vigilância em Saúde. SIVEP-Malária [Internet]. 2014 [cited 20 Sep 2017]. Available: http://portalsaude.saude.gov.br/index.php/o-ministerio/principal/leia-mais-o-ministerio/662-secretaria-svs/vigilancia-de-a-a-z/malaria/11346-situacao-epidemiologica-dados

[22] World Health Organization. Preterm birth [Internet]. 2017 [cited 12 Apr 2018] p. 1. Available: http://www.who.int/mediacentre/factsheets/fs363/en/

[23] de Camargo KR Jr., Coeli CM. Reclink: aplicativo para o relacionamento de bases de dados, implementando o método probabilistic record linkage. Cad Saude Publica. 2000;16: 439–447. doi: 10.1590/S0102-311X2000000200014 10883042 10.1590/s0102-311x2000000200014

[24] Hosmer DW, Lemeshow S. Applied Logistic Regression 2nd Ed. New York: Wiley; 2013.

[25] Albiti AH, Adam I, Ghouth AS. Placental malaria, anaemia and low birthweight in Yemen. Trans R Soc Trop Med Hyg. 2010;104: 191–4. doi: 10.1016/j.trstmh.2009.07.004 19716578 10.1016/j.trstmh.2009.07.004

[26] Bardají A, Sigauque B, Sanz S, Maixenchs M, Ordi J, Aponte JJ, et al Impact of malaria at the end of pregnancy on infant mortality and morbidity. J Infect Dis. 2011;203: 691–9. doi: 10.1093/infdis/jiq049 21199881 10.1093/infdis/jiq049 PMC3071276

[27] Valea I, Tinto H, Drabo MK, Huybregts L, Sorgho H, Ouedraogo J-B, et al An analysis of timing and frequency of malaria infection during pregnancy in relation to the risk of low birth weight, anaemia and perinatal mortality in Burkina Faso. Malar J. BioMed Central Ltd; 2012;11: 71 doi: 10.1186/1475-2875-11-71 22433778 10.1186/1475-2875-11-71 PMC3338396

[28] Carmona-Fonseca J, Maestre-B A. Incidencia de las malarias gestacional, congénita y placentaria en Urabá (Antioquia, Colombia), 2005–2007. Rev Colomb Obstet Ginecol. 2009;60: 19–33. Available: http://www.scopus.com/inward/record.url?eid=2-s2.0-69549138563&partnerID=tZOtx3y1

[29] Bardají A, Martínez-Espinosa FE, Arévalo-Herrera M, Padilla N, Kochar S, Ome-Kaius M, et al Burden and impact of Plasmodium vivax in pregnancy: A multi-centre prospective observational study. PLoS Negl Trop Dis. 2017;11: 1–22. doi: 10.1371/journal.pntd.0005606 28604825 10.1371/journal.pntd.0005606 PMC5481034

[30] Parekh FK, Hernandez JN, Krogstad DJ, Casapia WM, Branch OH. Prevalence and risk of Plasmodium falciparum and P. vivax malaria among pregnant women living in the hypoendemic communities of the Peruvian Amazon. Am J Trop Med Hyg. 2007;77: 451–7. Available: http://www.ncbi.nlm.nih.gov/pubmed/17827359 17827359 PMC3773697

[31] de Almeida LB, Barbosa M das GV, Martinez-Espinosa FE. Malária em mulheres de idade de 10 a 49 anos, segundo o SIVEP-Malária, Manaus, Amazonas, 2003–2006. Rev Soc Bras Med Trop. 2010;43: 304–308. doi: 10.1590/S0037-86822010000300018 20563501 10.1590/s0037-86822010000300018

[32] Jarude R, Trindade R, Tavares-Neto J. Malária em grávidas de uma maternidade pública de Rio Branco (Acre, Brasil). Rev Bras Ginecol e Obs. 2003;25: 149–154. doi: 10.1590/S0100-72032003000300002

[33] Chagas ECDS, Do Nascimento CT, De Santana Filho FS, Bôtto-Menezes CH, Martinez-Espinosa FE. Malária durante a gravidez: efeito sobre o curso da gestação na região amazônica. Rev Panam Salud Pública. 2009;26: 203–208. 20058829 10.1590/s1020-49892009000900003

[34] Cottrell G, Moussiliou A, Luty AJF, Cot M, Fievet N, Massougbodji A, et al Submicroscopic Plasmodium falciparum Infections Are Associated With Maternal Anemia, Premature Births, and Low Birth Weight. Clin Infect Dis. 2015;60: 1481–1488. doi: 10.1093/cid/civ122 25694651 10.1093/cid/civ122

[35] Stanisic DI, Moore K a., Baiwog F, Ura a., Clapham C, King CL, et al Risk factors for malaria and adverse birth outcomes in a prospective cohort of pregnant women resident in a high malaria transmission area of Papua New Guinea. Trans R Soc Trop Med Hyg. 2015; 313–324 doi: 10.1093/trstmh/trv019 25758854 10.1093/trstmh/trv019 PMC6592412

[36] Hartman TK, Rogerson SJ, Fischer PR. The impact of maternal malaria on newborns. Ann Trop Paediatr. 2010;30: 271–282. doi: 10.1179/146532810X12858955921032 21118620 10.1179/146532810X12858955921032

[37] United Nations Children’s Fund and World Health Organization. Low Birthweight: Country, regional and global estimates 2004;

[38] Moore KA, Simpson JA, Wiladphaingern J, Min AM, Pimanpanarak M, Paw MK, et al Influence of the number and timing of malaria episodes during pregnancy on prematurity and small-for-gestational-age in an area of low transmission. BMC Med. BioMed Central; 2017;15: 117 doi: 10.1186/s12916-017-0877-6 28633672 10.1186/s12916-017-0877-6 PMC5479010

[39] Kalilani L, Mofolo I, Chaponda M, Rogerson SJ, Meshnick SR. The effect of timing and frequency of Plasmodium falciparum infection during pregnancy on the risk of low birth weight and maternal anemia. Trans R Soc Trop Med Hyg. Royal Society of Tropical Medicine and Hygiene; 2010;104: 416–22. doi: 10.1016/j.trstmh.2010.01.013 20207387 10.1016/j.trstmh.2010.01.013 PMC4844554

[40] Albrecht L, Castiñeiras C, Carvalho BO, Ladeia-Andrade S, Santos da Silva N, Hoffmann EHE, et al The South American Plasmodium falciparum var gene repertoire is limited, highly shared and possibly lacks several antigenic types. Gene. Elsevier; 2010;453: 37–44. doi: 10.1016/j.gene.2010.01.001 20079817 10.1016/j.gene.2010.01.001

[41] Brazilian Ministry of Health, Secretaria de Vigilância em Saúde. Importância da gota espessa nas consultas de pré-natal. In: Programa Nacional de Controle da Malária Coordenador Geral, Ministério da Saúde, Nota Técnica—CGPNCM/DIGES/SVS/MS [Internet]. Brasília; 2014 [cited 14 Aug 2017]. Available: http://189.28.128.100/dab/docs/portaldab/notas_tecnicas/nota_informativa_conjunta.pdf

