# Supplementary material for: Malaria during pregnancy and newborn outcome in an unstable transmission area in Brazil: A population-based record linkage study
Source: PLoS One. 2018 Jun 21;13(6):e0199415. doi: 10.1371/journal.pone.0199415 (PMC6013245; doi:10.1371/journal.pone.0199415)
Supplement: S1 Table — Data from the System Information of Live Births provided by the Cruzeiro do Sul Municipal Secretariat of Health. a Information generated based on the number of live births and deaths reported by the mother. b There are other groups with ignored values. (DOCX) [file pone.0199415.s001.docx]

# S1 Table. Characteristics of mothers and newborns per year in Cruzeiro do Sul, 2006-2014.

| Characteristics | 2006  (N=1594) | 2007  (N=1795) | 2008  (N=1748) | 2009  (N=1625) | 2010  (N=1382) | 2011  (N=1510) | 2012  (N=1553) | 2013  (N=1620) | 2014  (N=1660) |
| --- | --- | --- | --- | --- | --- | --- | --- | --- | --- |
| Age, years, mean (SD) | 23.6 (6.1) | 23.7 (6.1) | 24.4 (6.3) | 24.5 (6.3) | 24.3 (6.3) | 24.3 (6.5) | 24.1 (6.4) | 24.3 (6.4) | 24.4 (6.6) |
| Primigravida, no. (%) ^a^ | 551 (34.6) | 481 (26.8) | 534 (30.6) | 530 (32.6) | 520 (37.6) | 644 (42.7) | 632 (40.7) | 659 (40.7) | 688 (41.5) |
| Gestational age, no. (%) |  |  |  |  |  |  |  |  |  |
| 22-27 weeks | 1 (0.1) | 5 (0.3) | 3 (0.2) | - | - | 5 (0.3) | - | 1 (0.1) | 3 (0.2) |
| 28-31 weeks | 5 (0.3) | 2 (0.1) | 7 (0.4) | 2 (0.1) | 11 (0.8) | 22 (1.5) | 16 (1.0) | 9 (0.5) | 7 (0.4) |
| 32-36 weeks | 27 (1.7) | 34 (1.9) | 55 (3.1) | 68 (4.2) | 64 (4.6) | 157 (10.4) | 199 (12.8) | 164 (10.1) | 213 (12.8) |
| 37 weeks or more | 1561 (97.9) | 1754 (97.7) | 1683 (96.3) | 1555 (95.7) | 1307 (94.6) | 1326 (87.8) | 1338 (86.2) | 1446 (89.3) | 1437 (86.6) |
| Marital status, no. (%) ^b^ |  |  |  |  |  |  |  |  |  |
| Single | 1408 (89.1) | 1503 (84.3) | 1390 (81.0) | 1329 (83.3) | 1021 (75.3) | 516 (34.8) | 414 (26.8) | 378 (23.6) | 369 (22.3) |
| Married or cohabiting | 163 (10.3) | 257 (14.4) | 310 (18.1) | 252 (15.8) | 327 (24.1) | 960 (64.6) | 1120 (72.6) | 1199 (74.9) | 1272 (76.8) |
| Years of formal education, no. (%) ^b^ |  |  |  |  |  |  |  |  |  |
| No education | 616 (38.8) | 622 (34.9) | 248 (14.4) | 202 (12.7) | 130 (9.5) | 86 (5.8) | 66 (4.3) | 77 (4.8) | 62 (3.8) |
| 1-3 years | 120 (7.6) | 135 (7.6) | 282 (16.4) | 239 (15.0) | 170 (12.5) | 168 (11.2) | 92 (6.0) | 63 (3.9) | 55 (3.4) |
| 4-7 years | 226 (14.2) | 264 (14.8) | 487 (28.3) | 469 (29.4) | 383 (28.1) | 390 (26.1) | 371 (24.0) | 317 (19.8) | 297 (18.1) |
| 8-11 years | 402 (25.3) | 295 (16.6) | 483 (28.1) | 477 (29.9) | 461 (33.9) | 675 (45.2) | 815 (52.7) | 914 (57.1) | 986 (60.2) |
| 12 or more | 222 (14.0) | 461 (25.9) | 219 (12.7) | 206 (12.9) | 217 (15.9) | 170 (11.4) | 196 (12.7) | 206 (12.9) | 215 (13.1) |
| Antenatal visits, no. (%) ^b^ |  |  |  |  |  |  |  |  |  |
| None | 245 (15.6) | 233 (13.1) | 295 (16.9) | 241 (15.0) | 118 (8.6) | 101 (6.7) | 57 (3.7) | 57 (3.5) | 34 (2.0) |
| 1-3 visits | 147 (9.4) | 132 (7.4) | 332 (19.1) | 399 (24.8) | 366 (26.7) | 285 (18.9) | 235 (15.1) | 213 (13.2) | 202 (12.2) |
| 4-6 visits | 216 (13.7) | 191 (10.8) | 583 (33.5) | 568 (35.4) | 552 (40.2) | 621 (41.1) | 591 (38.1) | 556 (34.3) | 543 (32.7) |
| 7 or more | 960 (61.1) | 1213 (68.3) | 523 (30.0) | 380 (23.7) | 314 (22.9) | 502 (33.3) | 669 (43.1) | 794 (49.0) | 881 (53.1) |
| Caesarean section, no. (%) | 317 (19.9) | 418 (23.3) | 469 (26.9) | 509 (31.3) | 543 (39.3) | 525 (34.8) | 623 (40.1) | 744 (45.9) | 752 (45.3) |
| Birth weight (g), mean (SD) |  |  |  |  |  |  |  |  |  |
| Mean (SD) | 3093 (523.6) | 3128 (529.0) | 3151 (506.7) | 3197 (509.6) | 3256 (524.0) | 3272 (503.7) | 3223 (515.0) | 3238 (486.2) | 3181 (521.9) |
| Median (IQR) | 3100 (2700-3450) | 3450 (2770-3480) | 3170 (2850-3500) | 3220 (2900-3515) | 3270 (2950-3590) | 3275 (2990-3585) | 3225 (2930-3560) | 3235 (2955-3538) | 3210 (2908-3500) |
| Low birth weight, no. (%) | 123 (7.7) | 134 (7.5) | 126 (7.2) | 111 (6.8) | 96 (7.0) | 80 (5.3) | 114 (7.3) | 95 (5.9) | 137 (8.3) |
| Very low birth weight, no. (%) | 6 (0.4) | 11 (0.6) | 10 (0.6) | 4 (0.3) | 4 (0.3) | 5 (0.3) | 7 (0.5) | 7 (0.4) | 17 (1.0) |

Data from the System Information of Live Births provided by the Cruzeiro do Sul Municipal Secretariat of Health.

^a^ Information generated based on the number of live births and deaths reported by the mother.

^b^ There are other groups with ignored values.
