# Supplementary material for: Malaria during pregnancy and newborn outcome in an unstable transmission area in Brazil: A population-based record linkage study
Source: PLoS One. 2018 Jun 21;13(6):e0199415. doi: 10.1371/journal.pone.0199415 (PMC6013245; doi:10.1371/journal.pone.0199415)
Supplement: S2 Table — N, number of malaria cases. Data are N or N (%). Values correspond to the total number of malaria episodes reported between 2006 and 2014. (DOCX) [file pone.0199415.s002.docx]

# S2 Table. Trends in malaria infection during pregnancy in Cruzeiro do Sul, 2006-2014.

| Cases of malaria | 2006 | 2007 | 2008 | 2009 | 2010 | 2011 | 2012 | 2013 | 2014 |
| --- | --- | --- | --- | --- | --- | --- | --- | --- | --- |
| Overall | 347 | 515 | 240 | 235 | 355 | 213 | 263 | 357 | 313 |
| *P. falciparum* | 140 (40.4%) | 139 (27.0%) | 72 (30.0%) | 51 (21.7%) | 80 (22.5%) | 46 (21.6%) | 90 (34.2%) | 143 (40.1%) | 122 (39.0%) |
| *P. vivax* | 201 (57.9%) | 370 (71.8%) | 166 (69.2%) | 183 (77.9%) | 274 (77.2%) | 167 (78.4%) | 172 (65.4%) | 214 (59.9%) | 188 (60.0%) |
| Mixed | 6 (1.7%) | 6 (1.2%) | 2 (0.8%) | 1 (0.4%) | 1 (0.3%) | 0 | 1 (0.4%) | 0 | 3 (1.0%) |

N, number of malaria cases. Data are N or N (%). Values correspond to the total number of malaria episodes reported between 2006 and 2014.
