# Supplementary material for: Malaria during pregnancy and newborn outcome in an unstable transmission area in Brazil: A population-based record linkage study
Source: PLoS One. 2018 Jun 21;13(6):e0199415. doi: 10.1371/journal.pone.0199415 (PMC6013245; doi:10.1371/journal.pone.0199415)
Supplement: S3 Table — N, number of individuals; IQR, interquartile range. a Malaria group consists of total pregnant women who had an infection (P. falciparum, P. vivax, and Mixed infections). b Differences between Non-Infected and the other groups were evaluated using Mann-Whitney rank sum tests. (DOCX) [file pone.0199415.s003.docx]

# S3 Table. Description of the birth weight of newborns from Non-Infected and Infected pregnant women per year.

|  | Non-infected | | Malaria ^a^ | | p value^b^ | *P. vivax* | | p value^b^ | *P. falciparum* | | p value^b^ | Mixed | | p value^b^ |
| --- | --- | --- | --- | --- | --- | --- | --- | --- | --- | --- | --- | --- | --- | --- |
| Year | N | Median (IQR) | N | Median (IQR) |  | N | Median (IQR) |  | N | Median (IQR) |  | N | Median (IQR) |  |
| 2006 | 1459 | 3100 (2700-3450) | 135 | 3030 (2660-3370) | 0.174 | 72 | 3135 (2770-3455) | 0.071 | 51 | 3005 (2615-3300) | 0.623 | 12 | 2828 (2613-3350) | 0.259 |
| 2007 | 1581 | 3170 (2795-3500) | 214 | 3078 (2690-3400) | 0.047 | 147 | 3100 (2720-3445) | 0.245 | 38 | 3018 (2640-3300) | 0.816 | 29 | 3060 (2500-3340) | 0.087 |
| 2008 | 1586 | 3190 (2860-3500) | 162 | 3000 (2730-3390) | 0.270 | 99 | 3100 (2770-3405) | 0.521 | 45 | 2990 (2600-3165) | 0.482 | 18 | 3055 (2770-3300) | 0.338 |
| 2009 | 1517 | 3230 (2910-3530) | 108 | 3118 (2850-3418) | 0.559 | 83 | 3210 (2880-3460) | 0.826 | 22 | 2900 (2750-3000) | 0.664 | 3 | 2660 (2415-3200) | 0.102 |
| 2010 | 1239 | 3300 (2970-3610) | 143 | 3090 (2785-3385) | 0.108 | 110 | 3095 (2790-3430) | 0.033 | 24 | 3185 (2995-3343) | 0.362 | 9 | 2630 (2420-2920) | 0.002 |
| 2011 | 1394 | 3288 (3010-3600) | 116 | 3123 (2780-3420) | 0.009 | 93 | 3150 (2800-3425) | 0.008 | 18 | 2973 (2670-3245) | 0.472 | 5 | 2880 (2760-3265) | 0.133 |
| 2012 | 1442 | 3233 (2930-3560) | 111 | 3100 (2845-3475) | 0.107 | 73 | 3200 (2870-3480) | 0.629 | 30 | 3030 (2745-3340) | 0.463 | 8 | 3075 (2865-3190) | 0.198 |
| 2013 | 1461 | 3245 (2975-3540) | 159 | 3145 (2860-3520) | 0.091 | 71 | 3210 (2810-3575) | 0.181 | 74 | 3128 (2890-3475) | 0.429 | 14 | 3270 (2920-3485) | 0.772 |
| 2014 | 1525 | 3215 (2915-3510) | 135 | 3160 (2855-3400) | 0.087 | 72 | 3063 (2805-3395) | 0.067 | 48 | 3205 (2958-3410) | 0.932 | 48 | 3300 (3140-3470) | 0.715 |

N, number of individuals; IQR, interquartile range.

^a^ Malaria group consists of total pregnant women who had an infection (*P. falciparum*, *P. vivax,* and Mixed infections).

^b^ Differences between Non-Infected and the other groups were evaluated using Mann-Whitney rank sum tests.
