# Supplementary material for: Malaria during pregnancy and newborn outcome in an unstable transmission area in Brazil: A population-based record linkage study
Source: PLoS One. 2018 Jun 21;13(6):e0199415. doi: 10.1371/journal.pone.0199415 (PMC6013245; doi:10.1371/journal.pone.0199415)
Supplement: S4 Table — N, number of individuals; no., number of newborns with low birth weight. a Malaria group consists of total pregnant women who had an infection (P. falciparum, P. vivax, and Mixed infections). b Differences between Non-Infected and Infected groups were evaluated using Chi-square tests. (DOCX) [file pone.0199415.s004.docx]

# S4 Table. Description of term low birth weight newborns from Non-Infected and Infected pregnant women per year.

|  | Non-infected | | Malaria ^a^ | | p value^b^ | *P. vivax* | | p value^b^ | *P. falciparum* | | p value^b^ | Mixed | | p value^b^ |
| --- | --- | --- | --- | --- | --- | --- | --- | --- | --- | --- | --- | --- | --- | --- |
| Year | N | no. (%) | N | no. (%) |  | N | no. (%) |  | N | no. (%) |  | N | no. (%) |  |
| 2006 | 1429 | 82 (5.7) | 132 | 13 (9.9) | 0.059 | 72 | 9 (12.5) | 0.019 | 49 | 3 (6.1) | 0.910 | 11 | 1 (9.1) | 0.635 |
| 2007 | 1551 | 91 (5.9) | 203 | 16 (7.9) | 0.259 | 141 | 10 (7.1) | 0.557 | 37 | 3 (8.1) | 0.568 | 25 | 3 (12.0) | 0.199 |
| 2008 | 1528 | 81 (5.3) | 155 | 10 (6.5) | 0.546 | 97 | 6 (6.2) | 0.707 | 40 | 4 (10.0) | 0.195 | 18 | 0 | 0.316 |
| 2009 | 1449 | 80 (5.5) | 106 | 4 (3.8) | 0.442 | 82 | 2 (2.4) | 0.228 | 22 | 1 (4.6) | 0.842 | 2 | 1 (50.0) | 0.006 |
| 2010 | 1175 | 57 (4.9) | 132 | 7 (5.3) | 0.820 | 105 | 5 (4.8) | 0.967 | 19 | 0 | 0.325 | 8 | 2 (25.0) | 0.009 |
| 2011 | 1229 | 32 (2.6) | 97 | 5 (5.2) | 0.142 | 77 | 3 (3.9) | 0.496 | 15 | 1 (6.7) | 0.330 | 5 | 1 (20.0) | 0.016 |
| 2012 | 1247 | 53 (4.3) | 91 | 5 (5.5) | 0.574 | 63 | 5 (7.9) | 0.165 | 22 | 0 | 0.323 | 6 | 0 | 0.606 |
| 2013 | 1306 | 38 (2.9) | 140 | 9 (6.4) | 0.026 | 60 | 4 (6.7) | 0.099 | 67 | 5 (7.5) | 0.037 | 13 | 0 | 0.533 |
| 2014 | 1322 | 67 (5.1) | 115 | 5 (4.4) | 0.734 | 59 | 5 (8.5) | 0.249 | 42 | 0 | 0.135 | 14 | 0 | 0.387 |

N, number of individuals; no., number of newborns with low birth weight.

^a^ Malaria group consists of total pregnant women who had an infection (*P. falciparum*, *P. vivax,* and Mixed infections).

^b^ Differences between Non-Infected and Infected groups were evaluated using Chi-square tests.
