# Supplementary material for: Malaria during pregnancy and newborn outcome in an unstable transmission area in Brazil: A population-based record linkage study
Source: PLoS One. 2018 Jun 21;13(6):e0199415. doi: 10.1371/journal.pone.0199415 (PMC6013245; doi:10.1371/journal.pone.0199415)
Supplement: S5 Table — N, number of individuals; SD, standard deviation; IQR, interquartile range. a Malaria group consists of total pregnant women who had an infection (P. falciparum, P. vivax, and Mixed infections). b Differences between each group were examined using Mann-Whitney or Kruskal-Wallis test with Dunn post hoc test. c p = 0.025 for P. vivax versus P. falciparum groups. (DOCX) [file pone.0199415.s005.docx]

# S5 Table. Description of birth weight of term newborns from Non-Infected and Infected pregnant women per gravidity.

| Birth weight (g) | Non-infected  (N=12,236) | Malaria ^a^  (N=1,171) | p value^b^ | *P. vivax*  (N=756) | p value^b^ | *P. falciparum*  (N=313) | p value^b^ | Mixed  (N=102) | p value^b^ |
| --- | --- | --- | --- | --- | --- | --- | --- | --- | --- |
| Primigravida |  |  |  |  |  |  |  |  |  |
| Mean (SD) | 3184 (454.8) | 3072 (453.1) | <0.0001 | 3088 (478.0) ^c^ | 0.0001 | 3022 (370.9) | 0.0003 | 3070 (436.6) | 0.104 |
| Median (IQR) | 3187 (2905-3475) | 3075 (2769-3355) |  | 3085 (2770-3370) |  | 3000 (2769-3260) |  | 3160 (2720-3348) |  |
| Multigravida |  |  |  |  |  |  |  |  |  |
| Mean (SD) | 3265 (494.5) | 3173 (477.2) | <0.0001 | 3199 (486.4) ^c^ | 0.0009 | 3137 (469.5) | <0.0001 | 3108 (424.1) | 0.003 |
| Median (IQR) | 3270 (2970-3585) | 3175 (2860-3460) |  | 3210 (2870-3500) |  | 3095 (2860-3420) |  | 3150 (2800-3340) |  |
| p value (Primigravida x Multigravida) | <0.0001 | 0.0004 |  | 0.0009 |  | 0.083 |  | 0.862 |  |

N, number of individuals; SD, standard deviation; IQR, interquartile range.

^a^ Malaria group consists of total pregnant women who had an infection (*P. falciparum*, *P. vivax,* and Mixed infections).

^b^ Differences between each group were examined using Mann-Whitney or Kruskal-Wallis test with Dunn post hoc test.

^c^ p=0.025 for *P. vivax* versus *P. falciparum* groups.
